# 肺癌抗血管生成治疗：现状、进展与困惑

**DOI:** 10.3779/j.issn.1009-3419.2022.101.16

**Published:** 2022-04-20

**Authors:** 舟 江, 建华 陈

**Affiliations:** 410006 长沙，中南大学附属肿瘤医院/湖南省肿瘤医院胸内一科 Department of Thoracic Medicine, Affiliated Cancer Hospital of Xiangya School of Medicine, Central South University, Hunan Cancer Hospital, Changsha 410006, China

**Keywords:** 肺肿瘤, 抗血管生成, 联合治疗, 新策略, Lung neoplasms, Anti-angiogenesis, Combination therapy, New strategy

## Abstract

肺癌是一种高度血管化的肿瘤，过去十多年，抑制血管生成已被证实是一种有效且极具前景的联合治疗选择，关于抗血管生成治疗与化疗、靶向治疗、免疫治疗的联合疗效数据在不断更新，其在晚期肺癌不同群体以及疾病的不同阶段均可带来获益，伴随而来的是对众多治疗策略的“排列组合”及“排兵布阵”的困惑，本文将基于临床现状及尚未解决的问题，结合最新临床及转化研究数据，对当前肺癌抗血管生成治疗作一综述。

## Abstract (en)

Lung cancer is a highly vascular tumors, over the past ten years, anti-angiogenes is has been proved to be an effective and highly promising combinational treatment. The data of the combination of anti-angiogenesis with chemotherapy, targeted therapy, immunotherapy has been constantly updating. Advanced lung cancer patients, no matter different groups or different stages of the disease, are benefited from anti-angiogenes. In this paper, based on the clinical status and unsolved problems, combined with the latest clinical and translational research data, we reviewed the current anti-angiogenesis treatment of lung cancer.

2004年，抗血管生成治疗率先打破了晚期非小细胞肺癌（non-small cell lung cancer, NSCLC）患者只有化疗的困局，使中位总生存期（overall survival, OS）首次突破1年，随后的分子靶向治疗、免疫治疗使晚期NSCLC治疗策略“百花齐放”，但抗血管生成治疗“历久弥新”，在不同治疗模式及治疗阶段均显示出联合效应。十余年的临床研究及真实世界数据证实，抗血管生成治疗联合化疗是经典模式，联合免疫检查点抑制剂可扩大免疫治疗获益人群，一线与靶向治疗联合可进一步提高生存期，且在不同亚组展现优势差异。当前认为这些协同效应的理论依据在于：①抗血管生成使局部血管正常化，促进化疗药物渗入乏氧的肿瘤中心，杀伤因“不可及”而产生耐药的肿瘤细胞^[1]^；②表皮生长因子受体酪氨酸激酶抑制剂（epidermal growth factor receptor tyrosine kinases inhibitor, EGFR-TKI）耐药的重要因素之一是下游通路激活，而血管内皮生长因子（vascular endothelial growth factor, VEGF）与EGFR具有相同的下游通路，联合抗VEGF可延缓EGFR-TKI耐药^[2]^；③VEGF抑制治疗使血管正常化，增加淋巴细胞浸润和T细胞活化^[3]^，此外，可加速形成高内皮性小静脉（high endothelial venules, HEV）^[10]^，通过介导向次级淋巴器官的迁移，增强循环B和T细胞的激活，当被密集的B和T细胞包围时，HEV可以进一步适应三级淋巴结构（tertiary lymphoid structure, TLS），从而触发强大的抗肿瘤免疫效应，显著改善患者的预后^[4]^。同时，随着“排列组合”的增多，肺癌抗血管生成治疗的临床决策又面临许多新的困惑，本文将就抗血管生成治疗在不同肺癌人群中的联合治疗现状、最新进展及需解决的问题与研究方向进行系统综述。

## 1 与化疗联合

### 1.1 贝伐珠单抗

#### 1.1.1 一线治疗

“里程碑式”研究ECOG4599^[5]^证实，贝伐珠单抗+紫杉醇+卡铂治疗Ⅲb期/Ⅳ期非鳞NSCLC较单纯化疗延长2个月无进展生存期（progression-free survival, PFS）（12.3个月 *vs* 10.3个月，HR=0.79），欧洲人群为主的AVAiL研究^[6]^结果显示不同剂量组（7.5 mg, 15 mg），贝伐珠单抗在PFS及客观缓解率（objective response rate, ORR）上均有显著获益，但并未转化成OS优势，原因考虑为后线治疗的复杂性，但亚组分析显示亚裔人群在不同剂量组的中位PFS均优于总人群；全球Ⅳ期研究SAiL，中国患者亚组分析亦显示出优于总人群的OS（18.5个月 *vs* 14.6个月）^[7]^；国内开展了专门入组中国人为主的亚裔人群的BEYOND研究^[8]^，在ORR（54% *vs* 26%）、中位PFS（9.2个月 *vs* 6.5个月，HR=0.4）及中位OS（24.3个月 *vs* 17.7个月，HR=0.68）上全面获益，这一结果进一步证实联合贝伐珠单抗较单纯含铂双药化疗在一线治疗中的优势地位，并且以中国人为代表的亚裔人群似乎获益更多。但以上研究并未区分驱动基因突变情况，近来一项中国真实世界研究数据^[9]^显示，一线含贝伐珠单抗方案较不含的方案，无论在驱动基因突变阳性或阴性人群中均有PFS获益。

#### 1.1.2 化疗方案

众多证实贝伐珠单抗一线治疗获益的研究所采用的化疗方案不尽相同，包括紫杉醇+卡铂、白蛋白紫杉醇+卡铂、吉西他滨+顺铂以及非鳞NSCLC优选的培美曲塞（+顺铂/卡铂），Pointbreak研究^[10]^基于意向治疗人群分析显示，贝伐珠单抗联合培美曲塞+卡铂或紫杉醇+卡铂在疾病控制后进入维持治疗阶段比例相当（62% *vs* 64%）；另一项纳入29项临床研究的*meta*分析^[11]^显示无论化疗方案是否包含紫杉醇，贝伐珠单抗一线联合化疗均可为非鳞NSCLC患者生存带来获益。因此，当前并未有证据指导贝伐珠单抗的“最佳化疗拍档”。

#### 1.1.3 维持与跨线

一线疾病控制后，众多研究对维持模式进行了探索。对美国国立综合癌症网络（National Comprehensive Cancer Network, NCCN）病例数据回顾分析及ARIES研究^[12]^证实了贝伐珠单抗单药维持较不维持均未获得近6个月的中位OS获益；AVAPERL研究^[13]^联合培美曲塞较贝伐珠单抗单药维持可进一步提高中位PFS及OS，但联合方案≥3级的副反应及严重不良事件（serious adverse event, SAE）发生率更高，COMPASS研究^[14]^亦证实联合培美曲塞较贝伐珠单抗单药显著延长PFS，EGFR野生型亚组分析提示联合方案中位OS显著延长（23.3个月 *vs* 18.3个月，HR=0.82）；后续基于高加索人群的ECOG5508^[15]^对贝伐珠单抗、培美曲塞、贝伐珠单抗+培美曲塞维持治疗疗效进行了头对头比较，结果显示联合维持方案并未比贝伐珠单抗单药延长中位OS，且3级-4级SAE发生率更高（51% *vs* 29%）。因此，当前单药或联合方案维持治疗，均有数据支持，但联合是否一定获益大过单药，并未有一致性结论，临床决策需综合考虑优势人群、肿瘤负荷及毒副反应。

Ⅲ期临床研究ULTIMATE及小样本回顾性研究^[16]^均证实一线单纯化疗进展后，二线或三线采用贝伐珠单抗联合化疗较多西他赛有显著生存获益，但基于当前临床现状，一线方案采用单纯化疗比例较低，免疫或靶向耐药后的抗血管生成二线治疗或者贝伐珠单抗跨线治疗的数据对临床指导作用更大。贝伐珠单抗在肠癌中的跨线治疗获益得到了证实，在肺癌领域，WJOG5610L研究及部分回顾性分析^[14, 17]^亦显示了贝伐珠单抗跨线在非鳞NSCLC中的PFS获益，但在后续的Ⅲ期研究AvaALL研究^[18]^中，跨线（二线及三线）治疗对比标准化疗的优势并未达到统计学差异（11.9个月 *vs* 10.2个月，*P*=0.104），可能与研究入组较慢、入组人数未达到预期、方案违背率较高有关。因此，贝伐珠单抗在NSCLC中的应用仍是较大的一个问号。

### 1.2 重组人血管内皮抑素

恩度是我国自主生产的重组人血管内皮抑素，相较于贝伐珠单抗的“做减法”，重组人血管内皮抑素是通过外源性补充血管生成的抑制分子。我国开展随机、双盲、对照、多中心研究^[19]^证实重组人血管内皮抑素联合长春瑞滨及顺铂较单纯化疗，可为经治及初治Ⅲ期/Ⅳ期NSCLC（包括鳞癌）带来近期疗效获益[中位疾病进展时间（time to progress, TTP）：6.3个月 *vs* 3.6个月]及生存质量改善。但并未有后续OS、维持及跨线治疗数据。

### 1.3 多靶点TKI

抗血管生成领域的多靶点TKI，通常在VEGF/VEGFR的基础上再覆盖血管生成其他通路及因子，如血小板源性生长因子/受体（platelet-derived growth factor/receptor, PDGF/PDGFR）、成纤维细胞生长因子/受体（fibroblast growth factor/receptor, FGF/FGFR）及C-KIT等。而多年来该类药物都止步于“低效高毒”，如一线索拉菲尼/凡德替尼联合化疗、二线索拉菲尼联合培美曲塞等，LUME-Lung1结果显示尼达尼布联合多西他赛二线治疗NSCLC较安慰剂有0.7个月PFS优势，腺癌亚组OS延长2.3个月，这一结果可作为该领域近十年的突破，但并未有后续进一步生存数据公布。安罗替尼亦作为我国自主研发的小分子多靶点TKI，显示出了明显优于同类药物的优势，原因可能归结为安罗替尼极低的半数抑制浓度（50% inhibitory concentration, IC_50_），在较低浓度即可发挥作用，较同类药物降低了毒副反应风险。基于ALTER0303研究，安罗替尼获批NSCLC三线治疗（较安慰剂中位OS延长3.3个月，中位PFS延长4个月）^[20]^，而其正在逐步挺进一线治疗，Ⅰ期研究显示联合化疗一线治疗驱动基因阴性NSCLC，30例患者的疾病控制率（disease control rate, DCR）达96.7%。一项*meta*分析发现安罗替尼较多个多靶点TKI在三线治疗中PFS优势更明显，因此，安罗替尼是该类药物中最具潜力的一员，并作为抗血管生成治疗，在临床研究中全面参与不同肺癌人群的一线治疗，但关于其与贝伐珠单抗的“一线优选”研究，并未有涉及，ALTER0303亚组分析提示既往贝伐珠单抗及重组人血管内皮抑素用药史对安罗替尼三线治疗的中位PFS及中位OS无影响^[20]^。

### 1.4 其他药物

雷莫芦单抗是全球第二个被批准用于NSCLC的大分子单抗，与贝伐珠单抗相比不同之处在于：①靶向VEGFR2；②适应证只有二线联合多西他赛；③因在鳞癌与非鳞癌中疗效相当，出血风险类似，成为第一个获批鳞癌适应证的抗血管生成药物^[21]^。但因药物可及性及在国内并未获批适应证，雷莫芦单抗应用较少，可作为后线个体化治疗的选择。

阿帕替尼作为我国首个自主研发的靶向VEGFR2的口服抗血管生成药物，Ⅱ期研究证实其三线治疗NSCLC较安慰剂显著提高ORR、DCR，明显延长mPFS，小样本探索性研究证实阿帕替尼联合培美曲塞及铂类一线治疗NSCLC，ORR和DCR分别达到80%和100%。

## 2 与免疫治疗联合

### 2.1 一线治疗

Impower150研究^[22]^较为全面地将免疫时代下，一线抗血管生成治疗需要比较的三种模式囊括，并且同时入组了*EGFR*/*ALK*敏感突变患者。最终的结果分析显示晚期非鳞NSCLC一线治疗，四药联合组（ABCP）中位OS超过25个月，在野生型患者，ABCP较ECOG4599模式（BCP）显著的OS获益（19.5个月 *vs* 14.7个月，HR=0.8），提示在抗血管生成联合化疗的基础上再加一个PD-L1抑制剂，可进一步提高生存获益；而阿特珠单抗联合化疗（ACP）相较贝伐珠单抗联合化疗（BCP）有4.3个月OS获益，但无统计学差异（*P*=0.05），对于这一结果，需考虑BCP组有46%患者至少接受了一次阿特珠单抗治疗，因此该组OS结果亦有免疫治疗因素。当前尚无法下结论一线免疫联合化疗优效于抗血管生成联合化疗，临床决策中或许仍需结合PD-L1等生物标志物综合考虑，此外，当前临床一线免疫联合方案，多为PD-1抑制剂，当前并未有PD-1抑制剂联合化疗与VEGF抑制剂联合化疗的头对头比较。我们可以确定的是免疫治疗与抗血管生成在晚期非鳞NSCLC一线治疗的协同效应。该研究另一个惊喜点在于突变人群的疗效，暂不讨论一线纳入敏感突变人群这一争议点，但结果显示在*EGFR*突变人群中，ABCP较BCP延长OS达11.3个月，降低死亡风险69%，虽亦未达到统计学差异，原因考虑较少的入组人数（26例 *vs* 32例）影响了效能，但明显分开的两条生存曲线，提示明显的生存获益，而ACP与BCP的生存数据近乎一致，综合证实抗血管生成治疗对于突变人群从免疫治疗中获益的关键作用，但过少的样本量及33%的不良反应终止治疗比例，让ABCP模式没能改变*EGFR*突变人群的一线治疗选择谱，但却为后续将“免疫+抗血管生存”作为突变人群耐药后治疗探索提供了依据和方向。另外肝转移亚组中也呈现了一样的结果和趋势：ABCP较BCP可显著延长生存，但ACP与BCP无明显差异，提示加入抗血管生成治疗后对免疫微环境的改善作用。2021年美国临床肿瘤学会（American Society of Clinical Oncology, ASCO）展示了一项单臂Ⅱ期研究，将ABCP中的化疗方案换成了培美曲塞+卡铂，同样入组突变人群，1年OS率达82.9%，似乎是在为四药联合寻找“最佳化疗搭档”，国内亦有类似的卡瑞利珠单抗联合阿帕替尼及白蛋白紫杉醇的“ABC”模式探索。

IMPOWER150展示出“免疫+抗血管生成”的巨大潜力，众多研究开始探索驱动基因阴性患者一线“去化疗”模式：Ⅰ期研究显示信迪利单抗联合安罗替尼DCR达100%^[23]^；卡瑞利珠单抗联合阿帕替尼Ⅱ期研究结果更新显示ORR: 40.0%，DCR: 92.0%，中位PFS达11.0个月，所有PD-L1表达水平亚组的疗效一致^[24]^；另一些PD-1及VEGF的双抗临床研究亦在开展中；但验证帕博利珠单抗联合仑伐替尼一线疗效的Ⅲ期研究LEAP-007以OS劣于安慰剂的阴性结果告终，提示多靶点抗血管生成TKI肺癌中的应用需谨慎。此外，笔者认为从“四药联合”到“去化疗”，仍需考虑肿瘤负荷并结合生物标志物。

### 2.2 跨线及后线探索

相较于“四药联合”及“去化疗”，当前临床晚期NSCLC一线免疫联合化疗应用更为广泛，如何让免疫联合抗血管生成在患者全程治疗中持续获益是另一个探索方向。2021年世界肺癌大会上公布了一项开放标签、两阶段、Ⅱ期研究评价贝伐珠单抗联合阿替利珠单抗治疗既往阿替利珠单抗单药治疗后进展的NSCLC患者的应用效果，旨在探索免疫跨线加用抗血管生成的疗效。类似的“再挑战”研究也在进行中，如帕博利珠单抗联合雷莫芦单抗对免疫治疗进展患者的疗效与安全性研究^[25]^等。另有一项Ⅱ期研究采用纳武利尤单抗+伊匹单抗联合尼达尼布治疗复发性NSCLC，无论前线治疗方案，旨在探索双免联合抗血管生成多靶点TKI的后线疗效。

## 3 与EGFR-TKI联合

### 3.1 一线治疗

从JO25567^[26]^到NEJ026^[27]^，贝伐珠单抗联合厄洛替尼较厄洛替尼单药显著延长PFS，奠定了“A+T”模式作为晚期*EGFR*突变人群一线优选的基础，但两项研究后续的OS数据（46个月-50个月）均未达到统计学差异，对于明显的PFS优势未转化为OS优势的原因仍需考虑复杂多样的后线治疗，且NEJ026研究中厄洛替尼单药组有28.6%患者进展后选择了贝伐珠单抗，此外，“A+T”模式下明显延长的生存期使JO2557失访率达16%。进一步的亚组分析显示了21L858R组获益更大，2021年ARTEMIS最新数据^[28]^显示联合治疗给21L858R亚组带来近10个月的PFS延长（19.5个月 *vs* 9.7个月），这也是目前抗血管联合EGFR-TKI治疗前瞻性临床研究中最好的结果，抗血管生成治疗再次为21L858R人群的疗效带来了突破，但相较于抗血管生成对免疫疗效改善机制的易理解，“A+T”在21L858R人群及19del人群中疗效差异的理论依据尚未有转化研究阐明。一线直接联合奥希替尼，ORR达80%，总人群中位PFS达19个月，但21L858R亚族没有显示出明显的优势^[29]^；这也提示“A+T”模式中，一代和三代TKI的优势人群不尽相同，而联合抗血管生成治疗的背景下，一代序贯三代或一线给予三代药物，也是未来要解决的临床困惑；另有Ⅰ期研究^[30]^探索了贝伐珠单抗联合阿法替尼的一线疗效（RR=0.813, DCR=100%），二代TKI的优势在于对非经典突变人群的覆盖，因此，未来期待针对该类人群的“A+T”模式的Ⅲ期临床研究数据。

在靶向VEGFR2方面，ACTⅣE研究发现阿帕替尼联合吉非替尼一线治疗可为总人群带来7个月的中位PFS优势（13.7个月 *vs* 10.2个月），且19del亚组获益更大；RELAY研究^[31]^证实联合雷莫芦单抗较厄洛替尼单药可获得7个月的PFS优势（19.4个月 *vs* 12.4个月），在2021年世界肺癌大会上，RELAY研究公布联合方案在19del不同突变之间的PFS获益差异，似乎不常见突变（19.4个月 *vs* 13.9个月）优于常见突变（E746_A750del：mPFS：15.2个月 *vs* 9.9个月）；以上提示不同抗血管生成靶点，其优势人群亦不同，即便同一药物，我们仍要带着异质性的观点去继续挖掘当前认为的各类亚组；此外，旨在评估雷莫芦单抗联合三代TKI疗效的Ⅲ期研究TORG1833正在进行中，亦期待其亚组分析结果。

安罗替尼联合吉非替尼对比吉非替尼的Ⅲ期研究（FL-ALTER）仍在进行中，目前仅有多项Ⅰ期、Ⅱ期的单臂研究数据，DCR可达100%，PFS达13个月。安罗替尼多靶点的特征在“A+T”模式下，会为患者带来较VEGF单抗更优的疗效，或是提高小分子抑制剂相关副反应，以及在不同突变亚型中疗效差异值得期待。

ACTⅣE研究^[32]^瞄准*EGFR*敏感突变晚期NSCLC（包括鳞癌）一线治疗，联合治疗组取得了整体人群PFS的显著获益。亚组分析显示，不同于CTONG1509，19del获益更明显（HR: 0.67 *vs* 0.72），另外携带*TP53*外显子8突变的患者获益明显（HR=0.24）。

### 3.2 耐药后选择及跨线治疗

在免疫治疗时代及“A+T”模式到来之前，贝伐珠单抗联合化疗可成为EGFE-TKI治疗失败后的选择，一项前瞻性、多中心、单臂Ⅱ期研究^[33]^数据显示入组30例患者，ORR及DCR分别为37%、83%，中位PFS及OS分别为6.6个月及18.2个月。在免疫治疗背景下，如何让突变人群在耐药后从免疫治疗中获益，是当下热点与难点，基于IMPOWER150的数据提示，“四药联合”或“免疫+抗血管生成”成为该领域探索模式，ORIENT-31是全球首个证实四药联合模式在EGFR-TKI耐药后疗效的Ⅲ期研究^[34]^，在2021年欧洲肿瘤内科学会（European Society for Medical Oncology, ESMO）上公布了结果，信迪利单抗+国产贝伐珠单抗类似物+培美曲塞+顺铂组（6.9个月）较贝伐珠单抗类似物+化疗组（5.6个月）及单纯化疗组（4.3个月）显著延长中位PFS，该研究同时入组T790M阳性、T790M阴性、一线使用一代/二代/三代EGFR-TKI、一代/二代耐药后序贯三代EGFR-TKI患者，证实“国产四药联合”模式在EGFR-TKI耐药后的广泛疗效；同时众多“去化疗”模式的临床研究正在进行中，卡瑞利珠单抗+阿帕替尼的Ⅰ期/Ⅱ期研究也显示出对耐药人群的潜在获益：DCR为82.4%，中位PFS为5.9个月。

一线“A+T”模式的显著获益使得贝伐珠单抗在突变人群中的介入时机前移，因此，一线联合靶向药物，耐药后跨线联合免疫治疗是今后抗血管生成治疗在突变人群中的潜在治疗模式，而目前尚无数据显示跨线甚至全程应用抗血管生成治疗的获益。

### 3.3 与其他靶点TKI联合

在*EGFR*突变人群中的显著获益，引发了对其他驱动基因阳性患者一线联合抗血管生成治疗策略的思考。首先是ALK，当前无一代ALK联合方案的临床研究，二代ALK抑制剂阿来替尼联合贝伐珠单抗治疗ALK阳性的晚期NSCLC的Ⅰ期/Ⅱ期研究（NCT02521051）正在进行中。而当前在*EGFR*突变人群中探索的问题，亦将成为其余靶点治疗要面临的困惑。

## 4 肺鳞癌

由于组织学差异导致的出血风险，以贝伐珠单抗为代表的抗血管生成治疗一直无法惠及肺鳞癌患者，当前可用于肺鳞癌的抗血管生成药物有三种：国内尚不可及的雷莫芦单抗^[21]^、国产重组人血管内皮抑素（恩度）^[19]^以及可用于周围型无出血风险鳞癌患者的安罗替尼^[35]^。如前所述，仅重组人血管内皮抑素有一线联合化疗优于单纯化疗的国内多中心数据，雷莫芦单抗在国外有二线适应证，安罗替尼一线治疗的临床研究尚在进行中。笔者认为未来肺鳞癌的探索方向可分为两个方面：①新靶点的发掘：雷莫芦单抗（抗VEGFR2）及安罗替尼（多靶点TKI）因不同靶点而突破鳞癌适应证，基础研究领域从肿瘤免疫代谢微环境的角度鉴定了许多潜在鳞癌抗血管生成靶点；②与免疫治疗的“排兵布阵”：免疫治疗为长期只有放化疗的晚期肺鳞癌治疗带来了突破，而在非鳞NSCLC中，抗血管生存与免疫治疗的协同效应可否推及鳞癌患者，一线“四药联合”及疾病稳定后的“去化疗”维持等治疗模式，在鳞癌领域都是空白。

## 5 小细胞肺癌

对于小细胞癌肺癌的抗血管生成治疗，多年来不断有研究尝试：贝伐珠单抗一线联合依托泊苷/伊利替康+顺铂^[36]^，阿柏西普（抗血管生成融合蛋白药物）二线联合拓扑替康^[37]^，重组人血管内皮抑素联合依托泊苷+顺铂以及小分子抑制剂阿帕替尼、帕唑帕尼及索拉菲尼联合含铂化疗^[38]^，都因阴性结果、入组例数太少或微弱获益限制了进一步的扩大研究，直到Ⅱ期研究ALTER1202^[35]^，证实安罗替尼三线治疗化疗失败的小细胞肺癌患者，显著延长中位PFS（4.1个月 *vs* 0.7个月，HR=0.19）及中位OS（7.3个月 *vs* 4.9个月，HR=0.53）。2021年ASCO展示了安罗替尼联合化疗一线治疗广泛期小细胞肺癌，ORR及DCR达90%及100%，OS达15个月；安罗替尼联合AK105（PD-1单抗）二线治疗化疗进展后的小细胞肺癌患者，ORR达50%，PFS达4.7个月，在众多PD-1单抗折戟于小细胞肺癌的背景下，这一结果非常瞩目，再次让大家思考联合抗血管生成会否改变PD-1单抗在SCLC的败局；结合PD-L1单抗在小细胞肺癌免疫治疗领域的突破，PD-L1单抗+安罗替尼+含铂化疗一线治疗广泛期小细胞肺癌的Ⅲ期研究正在进行。

## 6 特殊人群

抗血管生成治疗脑转移、伴恶性胸腔积液、老年患者等临床上“特殊人群”也显示出一致获益。Brain研究^[39]^纳入91例无症状脑转移Ⅳ期非鳞NSCLC患者，证实了贝伐珠单抗+卡铂+紫杉醇对脑转移患者的一线疗效（中位PFS：6.7个月；中位OS：16个月）。回顾性研究^[40]^证实联合贝伐珠单抗较单纯培美曲塞+铂类化疗可明显提升肺腺癌伴脑转移患者的颅内PFS（24.3个月 *vs* 10.9个月）；一项回顾性分析及ALTER0303^[41, 42]^证实联合贝伐珠单抗二线及安罗替尼三线治疗无症状脑转移的获益；对于安全性，囊括多瘤种的探索性回顾性分析^[43]^显示贝伐珠单抗并未增加颅内出血风险，此外，对于放射性脑水肿及脑坏死患者，贝伐珠单抗较单纯激素治疗可显著提高有效率（66.5% *vs* 31.5%）及获益人数（62.1% *vs* 42.6%）。在突变人群中，CTONG-1509^[28]^证实，一线联合贝伐珠单抗较厄洛替尼单药可显著降低58%的脑转移风险；回顾性分析显示联合治疗在颅内ORR、全身ORR、颅内PFS、全身PFS及OS全面获益。对于伴恶性胸腔积液患者，日本一项Ⅱ期研究^[44]^胸腔积液中VEGF水平高低显著影响患者胸腔中位PFS及OS，中国的一项研究证实联合贝伐珠单抗胸腔灌注较单纯顺铂组显著提高胸水控制率（88.33% *vs* 50%）并降低胸腔积液VEGF表达。中国一项Ⅲ期研究^[45]^证实重组人血管内皮抑素联合顺铂较重组人血管内皮抑素或者顺铂单药组显著提高胸水ORR，且在血性胸水亚组，联合组及重组人血管内皮抑素单药组的ORR及TTP均显著优于顺铂单药组。此外，SAiL^[7]^、TORG1323^[46]^、ARIES^[12]^、ALTER0303^[20]^等多项研究均显示在不同年龄分组（65岁、70岁、75岁），联合抗血管生成治疗的获益并无差异，提示高龄并非贝伐珠单抗及安罗替尼的疗效的独立影响因素。

## 7 转化研究方向

针对以上提到的临床困惑，当前基础研究领域聚焦在以下几个方面：①利用肿瘤相关血管内皮细胞及正常血管内皮细胞之间的代谢差异发掘疗效与毒副反应的生物标志物：可加速糖酵解的PFKFB3，可募集胰蛋白酶阳性肥大细胞的MYC及脯氨酸从头生物合成必需的酶和胆固醇生物合成的限速酶^[47, 48]^等；②阐明肿瘤免疫微环境对血管生成及耐药的调控：如成纤维细胞及骨髓源性抑制细胞对细胞外基质的重塑作用，胰蛋白酶阳性肥大细胞（mast cells, MCs）的数量与微血管密度的线性相关性^[49, 50]^；③将靶向非血管生成方式（血管选定及血管生成拟态等）纳入策略制定范围^[51]^；④鉴定血管生成过程中的协同、耐药靶点，使抗血管生成不局限于抗VEGF：如酪氨酸激酶受体Tie1^[52]^、参与血管生成的保守肽Apelin^[53]^等；⑤借助新技术解决药物脱靶、肿瘤内部渗透度等问题：如构建针对纤连蛋白结构域EⅢB或TEM8（TEC标志物之一）的嵌合抗原受体T细胞治疗^[54]^、纳米材料^[55]^、靶向VEGF及PDGF的双特异性抗体^[56]^。

## 8 总结与展望

从含铂双药化疗，到分子靶向治疗，再到免疫治疗，晚期肺癌治疗格局在近十年迅速发生变化，而抗血管生成治疗贯穿始终，与每种治疗都具有协同效应，在不同人群（病理分型、分子分型）以及同一人群不同治疗阶段（一线、跨线、后线）均有获益，其高效的“辅助角色”毋庸置疑，但广泛的受益面也使得我们仍需探讨最佳联合治疗方案（四药联合、去化疗）、最佳给药模式（单抗、小分子抑制剂）以及以上方案和模式在不同亚组人群及不同治疗阶段的最佳“排兵布阵”；转化研究发掘新型治疗及耐药靶点、疗效与毒副反应生物标志物等将为抗血管生成治疗的进一步精准优化打下基础。
